# Surgical removal of vena cava filter located on the right ventricle associated with bilateral pulmonary thromboendarterectomy case report

**DOI:** 10.1186/1749-8090-8-S1-P56

**Published:** 2013-09-11

**Authors:** V Campagnucci, A Silva, E Chamlian, S Gandra, L Rivetti

**Affiliations:** 1 Cirurgia, Faculdade de Ciências Médicas da Santa Casa de São Paulo, Brasil

## Background

The use of vena cava filter has been broadly indicated for the prevention of pulmonary thromboembolism (PTE) in patients with contraindication for the use of anticoagulants, PTE recurrence during anticoagulation, prophylactic and temporary use in surgical patients with high risk of thromboembolism, and with contraindication for anticoagulation. This is a procedure of easy execution and low risk of complications. However, cases have been reported of the device migration to cardiac chambers with high morbidity and risk of death caused by arrhythmias, tricuspid valve insufficiency, cardiac tamponade, and cardiogenic shock. We are reporting the case of a patient submitted to neurosurgery who developed profound venous thrombosis immediately after surgery. Due to the formal contraindication for anticoagulation, she was submitted to a vena cava filter engraft.

## Methods

Three weeks after she was admitted at the emergency unit with complaints of recurrent episodes of palpitation, progressive dyspnea and low pulse oximetry. The chest computerized tomography showed signs of extensive bilateral pulmonary thromboembolism, and image consistent with vena cava filter on projection of the right ventricle. The echo-doppler-cardiogram showed the image of a mobile metallic structure at the exit of the right ventricle and mild tricuspid valve insufficiency. With this diagnose, the patient was submitted to open heart surgical treatment for extraction of the device, due to the tricuspid valve onset. Additionally, an extensive bilateral pulmonary thromboendarterectomy was performed.

## Results

There were no complications, and the patient was discharged from the hospital 10 days after surgery.

## Conclusion

The vena cava filter withdrawal may be performed by endovascular technique, or through surgery, the later being the most desirable when there is a tricuspid valve involvement. The direct visualization allowed for a careful removal of the device without aggravation of the preexistent injury.

